# Smart Cognitive IoT Devices Using Multi-Layer Perception Neural Network on Limited Microcontroller

**DOI:** 10.3390/s22145106

**Published:** 2022-07-07

**Authors:** Mahmoud Hussein, Yehia Sayed Mohammed, Ahmed I. Galal, Emad Abd-Elrahman, Mohamed Zorkany

**Affiliations:** 1 National Telecommunication Institute (NTI), 5 Mahmoud El Miligui Street, 6th District-Nasr City, Cairo 11768, Egypt; mah.hussein@nti.sci.eg (M.H.); m_zorkany@nti.sci.eg (M.Z.); 2 Faculty of Engineering, Minia University, Minia 61519, Egypt; yahia.ali@mu.edu.eg (Y.S.M.); galal@mu.edu.eg (A.I.G.)

**Keywords:** cognitive IoT, smart nodes, critical systems, neural networks, microcontrollers

## Abstract

The Internet of Things (IoT) era is mainly dependent on the word “Smart”, such as smart cities, smart homes, and smart cars. This aspect can be achieved through the merging of machine learning algorithms with IoT computing models. By adding the Artificial Intelligence (AI) algorithms to IoT, the result is the Cognitive IoT (CIoT). In the automotive industry, many researchers worked on self-diagnosis systems using deep learning, but most of them performed this process on the cloud due to the hardware limitations of the end-devices, and the devices obtain the decision via the cloud servers. Others worked with simple traditional algorithms of machine learning to solve these limitations of the processing capabilities of the end-devices. In this paper, a self-diagnosis smart device is introduced with fast responses and little overhead using the Multi-Layer Perceptron Neural Network (MLP-NN) as a deep learning technique. The MLP-NN learning stage is performed using a Tensorflow framework to generate an MLP model’s parameters. Then, the MLP-NN model is implemented using these model’s parameters on a low cost end-device such as ARM Cortex-M Series architecture. After implementing the MLP-NN model, the IoT implementation is built to publish the decision results. With the proposed implemented method for the smart device, the output decision based on sensors values can be taken by the IoT node itself without returning to the cloud. For comparison, another solution is proposed for the cloud-based architecture, where the MLP-NN model is implemented on Cloud. The results clarify a successful implemented MLP-NN model for little capabilities end-devices, where the smart device solution has a lower traffic and latency than the cloud-based solution.

## 1. Introduction

Now, we have a large number of IoT devices, many of them have limited processing and intelligence capacities while others are sophisticated enough to have these computing capacities. So, a lightweight communication protocol is required for the IoT communication phase. The message queuing telemetry transport (MQTT) protocol is one of the broadly used protocols in IoT messaging communication. It is the pub/sub protocol that outperforms traditional communication protocols such as HTTP as a very known client/server architecture. The preference of MQTT comparing to other protocols came from its low overhead in terms of packet size and transmission delay for real time applications as proven in [1].

Combining the aspects of artificial intelligence (AI), machine learning (ML), mathematical decision models, or deep-learning (DL) with IoT can lead to the cognitive IoT (CIoT) architecture. This classification had been considered in the literature review about the cognitive sensing for many smart applications [2]. Moreover, the integration of smart IoT modules in the critical applications have been considered such as transportation in oil lines control-based supervisory control and data acquisition (SCADA) systems [3] and automotive industry domain.

Normally, the vehicles’ diagnosis and checking are performed in the maintenance centers [4] and drivers should go to these centers to check for any problems found in their vehicles [5]. Vehicles have some indication alarms that can help drivers to know if there is a serious problem in the vehicle [6] and they should also do a vehicle check. Some of the drivers do not know what these alarms indicate, and they will go to the maintenance center for every alarm indicator resulting in wasted money and time if the alarm is not related to a malfunction in one or more of the vehicle parts. The CIoT framework is one of the modern solutions to overcome these issues [7]. For such a framework, this combination requires a kind of TinyML running over specific kinds of microcontrollers.

Tiny Machine Learning (TinyML) is a type of ML that is integrated with embedded systems to enable running ML models on low-power microcontroller devices. It is capable of performing AI applications on-device sensor data analytics using low-memory and low-power resources. The proposed system delivers intelligence/smart to low-power/low-memory microcontrollers (tiny devices) by enabling machine learning on these limited microcontrollers. A standard IoT device just collects data from sensors and sends it over the cloud via an IoT server, where the proposed smart IoT devices host machine-learning models. So the proposed device as a TinyML device can optimize the ML-model (MLP-NN) to work on limited resource- devices. It can eliminate and control the necessity of data transmission to the IoT server. It can also take decisions not just send data as the standard IoT device. It adds intelligence to millions of IoT devices that could be used every day.

This research can help drivers with a Smart CIoT Unit that helps in diagnosis and reduces the number of visits to the maintenance centers. It also encourages safe driving. To make an IoT device smart and help in diagnosis, the device should be able to learn and train with a machine learning technique to have artificial intelligence decision making.

To cope with these objectives, there are two methods to do the learning process: training the AI model on the cloud or on the device. In the first method, the learning and training process will be handled on the cloud, as well as the decision-making [8,9]. At running mode, based on this method, the device should gather the sensor readings and publish them to the cloud for decision-making, the cloud will make the decision based on the learning process and return the result or output back to the device. Although this method is simple, it has some disadvantages as the learning process is performed totally on the cloud, which implies a delay due to sending inputs to the cloud and waiting for output that should be sent back to the device. Another disadvantage, if the internet connection is disconnected for any reason, then the device is not able to make any decision that may be a critical one. The second method, the learning and training process will be handled on the device, as this device will be a microcontroller [10,11]. In this case the microcontroller must have high specifications from processor speed and memory. However, Most available microcontrollers are limited in resources, which implies limited learning with respect to the power cloud computing aspect.

In this paper, our proposal will consider the training process on the cloud, the weights’ matrix is output from this training process. An AI structure of a neural network is built on the device with the training outcomes, which helps the device to be a decision maker without the fear of a cloud disconnection. The proposed method overcomes the delay and latency problem of the first method and the limited learning problem in the second method due to the limited resources of the device. For comparison, another solution is proposed for the cloud-based architecture in automotive industrial applications, where the machine learning model is trained and implemented on the cloud. So, the decision making will be performed on the cloud. To conclude, the first method’s learning and decision making is all performed on the cloud while in the second method, it is all performed on a device with high performance capabilities. However, our proposals consider the training phase on the cloud while the decision making will be performed on a limited microcontroller.

The remainder of this work is structured as follows: In Section 2, we give an overview of the relevant work. In Section 3, we describe the structure of our proposed solution. Section 4 presents the implementation phase based on the selected hardware for our framework and considers the communication phase based on the MQTT protocol. The performance evaluation of our proposed architecture will be handled in Section 5. Finally, conclusions and future works are presented in Section 6.

## 2. Related Work

The ADAS system is designed to help drivers to prevent or reduce vehicle accidents and the serious impact of accidents that drivers cannot avoid it. As ADAS could be used in accident avoidance or detection, it is also used in many applications such as lane departure corrections, automatic parking, traffic sign recognition, blind spot detection, and other many applications to assist drivers and save lives. The current and future objectives of the ADAS systems are to; increase the system reliability, develop systems in shorter development cycles, and reduce cost to be able to be used in all vehicles. The proposed system is one of the ADAS applications to realize autonomous vehicles and validate these current and future objectives of the ADAS systems by adding smart decisions part based on machine learning models such as MLP-NN.

The MLP-NN concepts are used even for different areas of interest. An audience evaluation system was introduced in [12]. It studies a model for the teaching audience evaluation system based on the multilayer perceptron. Authors showed that the performance evaluation of symphony based on the MLP can be evaluated in real-time, and it is at least more accurate than the obtained results by the mainstream method of data post-processing with iterative algorithms 23.1%.

An improved authentication system was proposed in [13]. It is a biometric authentication system that uses the MLP-NN. Researchers propose an existing authentication mechanism improvement through using Keystroke Dynamics (KD), MLP-NN and the Most Valuable Player Algorithm (MVPA). To overcome the issues in the conventional training process, the MLP-NN model is trained using the MVPA. With MATLAB software, their proposed biometric authentication system is validated and developed on variant users.

Another work in [14] uses the MLP-NN modeling for a land prediction system. It uses images from a Landsat satellite to determine the land-usage. Their prediction model was validated using classified and simulated LULC maps of 2018 that are resulted into an overall-accuracy to 88%. Results indicate that a 389.27% increase in built-up area as the dominant land-use change in 1990–2018 and an increase of 56.25% of built-up area is forecasted in the years 2018–2040. The land consumption rate and land absorption coefficient indices are used to determine urban expansion. The observations derived from their research are useful because they will help regional planners to forecast land-usage.

A malicious traffic detection system was introduced by [15]. It introduced an AI-based solution for malicious traffic detection using MLP-NN. Authors explore the accuracy of the MLP-NN learning algorithm for detecting botnet traffic from the IoT devices that are infected by two main IoT botnets, namely, Bashlite and Mirai (also called Gafgyt). After optimization and tuning steps, the MLP-NN algorithm got an accuracy 100% during the testing phase of the IoT classification of the botnet traffic.

The Advanced Driver Assistance System (ADAS) visions introduced in this work [16] covered the whole of the issues in this domain. They considered different aspects and vital points of view regarding an ADAS system.

The work in [17] introduced an embedded system from development and assessment views. They evaluated some indicators in their overall assessment such as safety, comfort and economy. Moreover, they considered different styles of driving in order to study the effects on safety for all scenarios and draw correlations between driving styles and the considered indicators.

Regarding the hardware implementation for AI codes on microcontrollers, the work in [18] highlighted this issue based on backpropagation training. They confirmed through this implementation the required minimum time for specific application-based microcontrollers.

This work [19] triggered a vital issue in transportation safety by analyzing driver behavior during the driving journey. They considered the data inputs gathered from the driver using IoT-machine learning systems and then sent them to the cloud for processing and creating the suitable alarms for drivers to increase the level of safety by avoiding some accidents scenarios.

Another work [20] considered the safety issue based on driver data and behavior has been introduced under the umbrella of Autonomous Vehicles (AV) through the Driver Assistance System (DAS). Their system can introduce different alerts to the driver based on machine learning algorithm outputs.

For the Driver Assistance System (DAS), the work in [21] introduced an Advanced DAS (ADAS) framework to consider safety and comfort indicators for the drivers. The prototype depends on using some ML tools to read some signs and messages and convert them into speech in order to help driver decisions. The work achieved reasonable accuracy based on the studied datasets.

In the same context of ADAS, the work in [22] considered the design of autonomous vehicles based on a generative adversarial network (GAN). Their design considered the autonomous one driving and the human based one by recommending everything through the driver car monitor.

This ADAS work [23] provides real-time object detection based on deep learning and considering specific CPUs to avoid the high price GPUs units in the current generation cars. They used specific NVIDIA series in their ADAS system to prove the detection efficiency compared to similar GPU units used by other countries, proving it is a cost-wise solution.

There are many research studies on Self-Diagnosis Systems [24,25]. The End-Devices for these systems have hardware limitations to do the full deep learning, so most of them did the processing on the Cloud. On the other hand, some of these studies worked with simple machine learning algorithms on the end-devices due to their limited capabilities. This research emphasis on deep learning with the hardware-limited end-devices does the learning process using a software utility, and then uses the output weights to make the end-device able to take the decision for any values for inputs.

An improved self-diagnosis system for autonomous vehicles based on the deep learning technique is introduced in [26]. This self-based system for cars can enhance the overall life cycle of the cars spare parts and the performance of self-diagnosis systems in terms of the self awareness model. The work considered the data processing inside the vehicle by an integrated module through gathering and processing the sensors data locally inside the car. The learning model in this proposal is cloud based. The activation functions used in this work are ReLU and the Sigmoid functions. They also proposed an edge computing model for exchanging the self-diagnosis information between cars through a cloud-based server. The work simulations are based on specific PCs not real IoT nodes or controllers as we consider in our work.

Another work in the direction of self-diagnosis systems had been proposed in [27]. A decision-making tree is used based on some specific measures in the vehicle for safety-based solutions considering server-less decisions. They proposed a machine learning-based technique called lightweight autonomous vehicle self-diagnosis (LAVS). Their proposal mainly focused on the communication protocols enhancement in such kinds of systems.

Cloud computing was introduced by many researchers and vendor-specific solutions to run some computational tasks in the cloud. While cloud computing has many benefits such as elasticity and scalability in computational models, IoT data transfer to and from the cloud is still a challenge in this direction, especially for real-time actions. The authors in [28] tried to propose some offloading mechanisms to overcome such latency problems to and from cloud data transfer. They proposed some kinds of task allocation strategies that are able to optimally select the best resource combination of computation and storage needed to execute some IoT smart tasks. It is a kind of resources reservation by grouping some tasks in order to execute and achieve real-time behavior. Such a direction is focused on spontaneous resources in terms of computation and storage consumption while keeping the lowest cost as an objective.

We can conclude the related work as shown in Table 1. Many researchers trained the MLP-NN models On-Cloud, in addition to the operation process and the decision-making being On-Cloud. Other studies trained and tested the MLP-NN models On-Device, as well as the operation process and the decision-making being On-Device. In the proposed solution, the training process is performed On-Cloud, and the operation process and the decision-making are performed On-Device.

## 3. Proposed Solutions

This section highlights the proposed solutions as follows:

### 3.1. Proposed Solutions Overview

**Figure 1:**
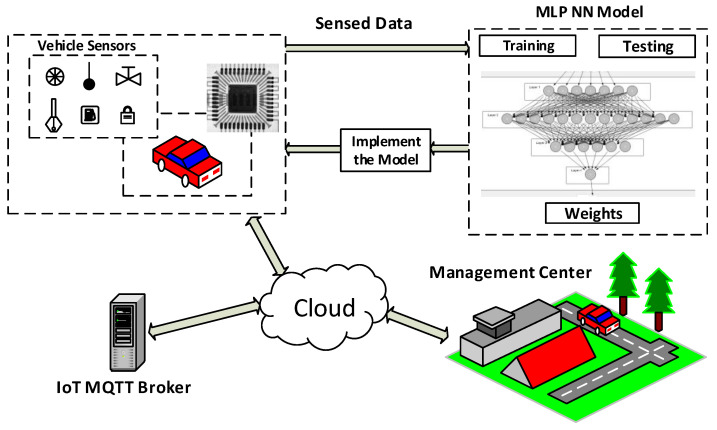
The proposed smart device architecture.

**Figure 2:**
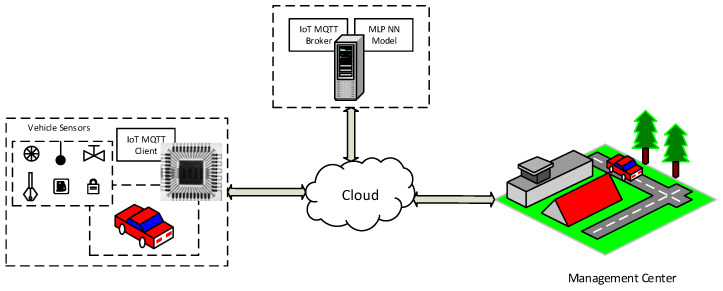
The proposed cloud-based architecture.

Two solutions are proposed and studied for the automotive industrial domain. The first solution is the cloud-based solution, where the decision maker is the cloud-based AI service. When the decision maker is taken by the device, this is the second solution. In this research, a study is conducted for the two solutions. As shown in Figure 1, the AI-Service is carried out with a cloud-based server to make the decision for the sensor data. In the smart device solution as shown in Figure 2, the device has locally implemented the MLP neural network model to make the decision by itself instead of sending data to the cloud to handle the decision-making process, so, an AI concept should be applied.

Therefore, a diagnostic system is proposed using neural networks to make a data analysis and to make suitable decisions. The proposed smart device sends the diagnostic problem or result directly to the management center instead of sending the data itself. This saves traffic and the time taken to obtain the final decision or the system status rather than the cloud-based solution. It includes two parts; the smart diagnosis device, and the IoT communication. The smart diagnosis device uses the MLP neural network to make a decision and knows the system status. For IoT communication, an MQTT broker is used to publish the result of the MLP-NN phase.

**Figure 3:**
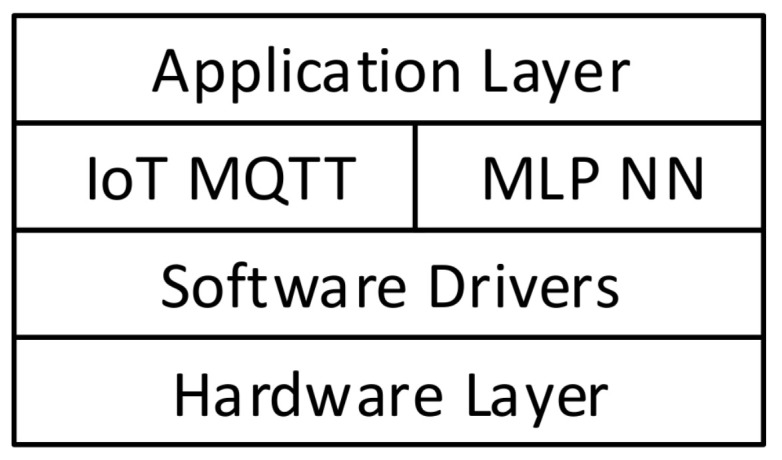
The layers stack for the proposed framework.

There are four layers for the software architecture as shown in Figure 3. The application layer has the implementation of the diagnosis system. The service layer has the IoT MQTT service and the MLP-NN service. The software drivers’ layer interfaces the hardware layer with the services and the application layer.

### 3.2. The Learning Smart Diagnostic Device Phase

At first, data-set was prepared with experts in the automotive field to include all the possibilities for the sensor readings. This variety in the data-set enhances the learning and testing phases. The data-set has six types of in-vehicle sensor readings (6 inputs); speed, obstacle distance, fuel level, engine temperature, oil level, and door Lock. Vehicle speed has four possibilities; stopped, slowly, normal, and exceed. Obstacle distances are zero, close, and normal distance. Fuel and oil levels, and the engine temperature have values of normal and abnormal. Four classification outputs (normal, small alarm, medium alarm, and big problem). Data was normalized for the best learning process.

**Figure 4:**
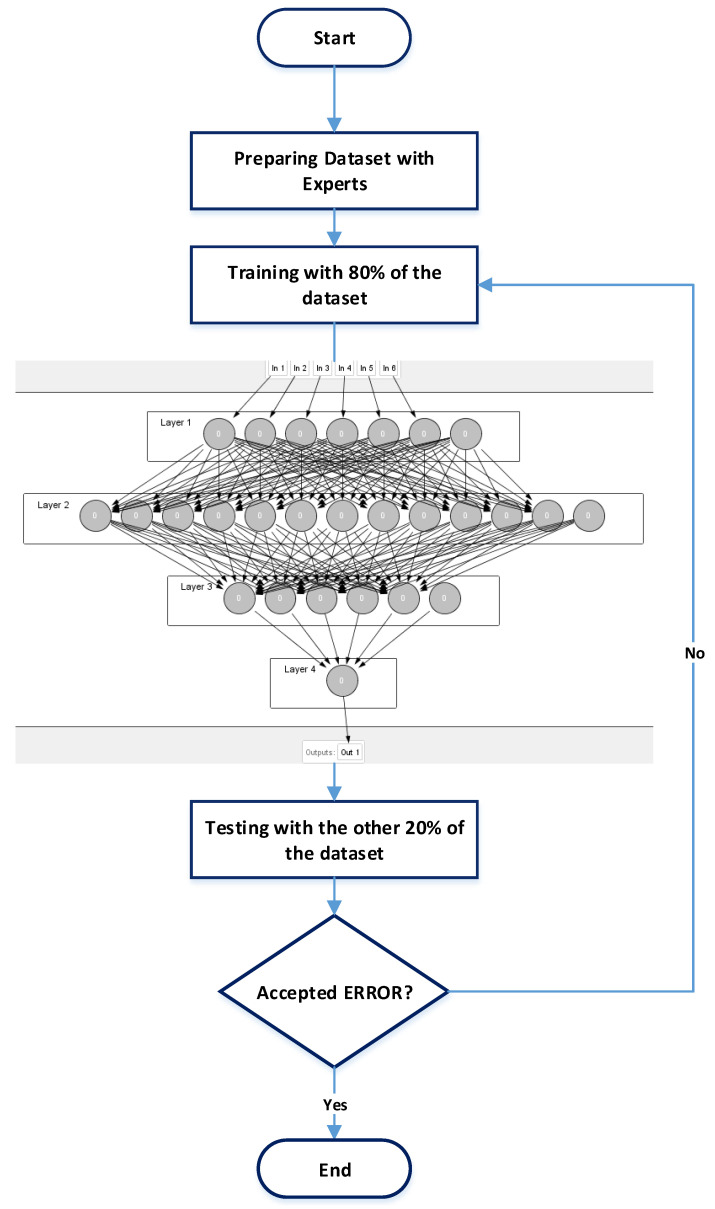
The learning process structure.

Figure 4 shows the general learning process structure of the proposed machine learning which depends on the MLP neural network model.

The training data is randomly selected from the data-set. It represents 80% of the data set. For the learning phase, the training data was taken as an input. The NN model is a multi-layer model, it consists of input layer, two hidden-layers, and one output layer as shown in Figure 4.

The input layer has six inputs; speed, obstacle distance, fuel level, engine temperature, Oil Level, and door Lock. The first hidden-layer composes of twelve neurons and the second hidden-layer composes of five neurons. Finally, the output layer has one neuron. The NN structure uses the Relu activation function that is shown in Figure 4. The training error reached 0.00001.

Finally, the testing phase is initiated with the testing data, which is the other 20% of the dataset. An error of less than 0.1 is accepted. If the error exceeds 0.1 for any test-case, the learning process will be repeated to reach less than 0.1 for all test-cases.

## 4. The Hardware Implementation of MLP-NN on the Cortex MCU

There are two ways to build a deep learning model on microcontrollers. The first method depends on standard ready-made frameworks such as TensorFlow Lite (developed at Google), Caffe framework (developed at University of California). The second method depends on building our model directly on the microcontrollers by a programming language such as Embedded C programming language.

Most AI developers have used the first ready-made method, which is a very easy method for AI developers who are not interested in programming embedded systems. However, this ready-made framework has many limitations to developing machine learning models on microcontrollers. For example; TensorFlow Lite is designed for the specific constraints of microcontrollers development (https://www.tensorflow.org/lite/microcontrollers, accessed on 1 June 2022). It supports limited subset operations of TensorFlow, limited sets of devices, and it requires manual memory management for Low-level C++ API. TensorFlow Lite for microcontrollers requires a 32-bit platform. It is limited to small number of microcontrollers such as ARM architecture of Cortex-M series, and ESP32. Moreover, some models are too big to store on microcontrollers. The optimization and efficiency in TensorFlow Lite is a trade-off on the model accuracy. Therefore, it has lower accuracy than their counterparts. Moreover, the Caffe deep learning framework has limitations on microcontroller types such as TensorFlow Lite. These ready-made frameworks can’t be used with low cost microcontrollers.

So, in our proposed design, we will use the second method which depends on building our model directly on the low cost and low power microcontrollers after training and testing the model using TensorFlow framework. Moreover, this type of microcontroller (ARM-Cortex) is the most available in the market.

### 4.1. Proposed AI Implementation on Limited Microcontroller

After the training process with the TensorFlow on the cloud, the weights matrix is obtained. Then, it will be taken as input to the MLP-NN software module that implements the MLP-NN Model on the MCU. As shown in the previous figure (Figure 4), the software should be initialized with the MLP-NN parameters such as the input count, weights matrix, MLP-NN layers, and the activation function used.

The MLP-NN layers are denoted as rounds. The software will iterate for every round. It starts with the first round as the current round. Then, it will calculate the new neurons for the next round with the selected activation function. It copies the next round to the current round to be calculated. The software will check if a new round is found. Then, it calculates the neurons of the next round until reaching the final round that represents the outputs. From these outputs, the decision is obtained.

After the training and testing process with the TensorFlow on the cloud, the weights matrix is obtained. Then, we can build the MLP-NN on the MCU using the same MLP-NN structure which is already built it in training phase, integrated with the weights matrix, and is obtained after the testing phase. It will be taken as input to the MLP-NN software module that implements the MLP-NN model on the MCU. Input neurons set *X* is defined as:

(1) $$X=\left(X_{1},X_{2},\ldots ,X_{n0},b\right)$$

where n0 is the number of input sensors in the input layer (layer 0), and *b* is the bias. The model neurons set *N* is defined as:

(2) $$N=\left(n^{0},n^{1},n^{2},\ldots ,n^{Z}\right)$$

where *Z* is the number of layers excluding the input layer. Weights $W^{L}$ that are obtained from the training process are defined as:

(3) $$W^{L}=\left\{\begin{array}{ccc} {W_{N_{1}}^{L-1}}_{N_{1}}^{L} & ........ & {W_{N_{1}}^{L-1}}_{N_{m}}^{L}\\ . & \; & .\\ . & \; & .\\ {W_{N_{n}}^{L-1}}_{{N_{1}}^{L}} & ........ & {W_{N_{n}}^{L-1}}_{{N_{m}}^{L}} \end{array}\right\}$$

where $W^{L}$ is the weight matrix for the layer *L*, ${W_{N_{1}}^{L-1}}_{N_{1}}^{L}$ is the weight of the link between the neuron $N_{1}$ in layer L−1 and the neuron $N_{1}$ in the layer *L*, *n* is the number of neurons for the layer L−1, *m* is the number of neurons for the layer *L*, and the ones column for bias. The weights set *W* is defined as following:

(4) $$W=w^{1},w^{2},\ldots ,w^{z}$$

The output layers neurons are obtained by applying the activation function ϕ as following:

(5) $$y^{L}=\phi \left(y^{L-1}W^{L}\right)$$

where $y^{L}$ is the output neurons vector for the layer *L*, $y^{L-1}$ is the output neurons vector for the previous layer L−1.

The Relu activation is used in neurons of hidden layers which is defined as:

(6) f(net)=max(0,net)

and

(7) $$net_{n}=W^{T}y^{\left(n-1\right)}$$

where *W* represents the weight parameters between the current NN layer and previous NN layer, $W^{T}$ is transpose of matrix *W*, and $y^{\left(n-1\right)}$ is the output of the previous NN layer. For the neuron of the output layer, the sigmoid activation function is used which is defined as:

(8) $$\phi \left(t\right)=\frac{1}{1+e^{-net}}$$

All output neurons set *Y* is defined as:

(9) $$Y=(y^{0},y^{1},y^{2},\ldots ,y^{Z})$$

where $y^{0}$ is the input neurons vector *X*, $y^{Z}$ is the output layer, and *Z* is the number of layers.

As shown in Algorithm 1, the software uses the MLP-NN structure that is used during the training phase with the obtained weights parameters *W*. The MLP-NN hyper-parameters such as the input layer neurons *X*, the weights parameters *W*, number of neurons for each layer, reference to the output neurons for all layers *Y*, and the number of layers *Z*. It acts for the input layer as a previous output $y^{0}$, then applies a multiplication of the neurons vector of this layer to the weight matrix $w^{1}$ that has the weights between the input layer (layer 0) and the next layer (layer 1). $y^{1}$ is calculated by taking the sigmoid activation function to every value obtained in the output vector. This behavior is repeated as shown in the algorithm for each layer in the MLP-NN model. Finally, the output layer $y^{Z}$ is obtained. The following pseudo code summarizes the previous steps.
**Algorithm 1: MLP-NN Model Decision Making****function**takeDecision(X,W,N,Y,Z)    Copy the input neurons *X* into the first output layer $y^{0}$in*Y*    **for** every layer *L* in range 1 to *z* (number of layers) **do**        Multiply the transpose of the layer L weights with the previous output layer: ${\left(W^{L}\right)}^{T}*y^{L-1}$        Calculate the ReLU activation function for the multiplication result: $\phi (y^{L-1}*W^{L})$        Copy the ReLU activation function results into the Layer L output $y^{L}$    **end for**    Obtain the decision from the last output layer $y^{Z}$**end function**

### 4.2. Implementation Steps

The embedded systems industry requires low-cost high-performance microcontrollers that do the specific system tasks. In this direction, the Cortex-M based microcontroller has few kilobytes to megabytes of flash memory (FLASH) and the static ram memory (SRAM). In addition to the limitation in the processing capability that is less than or equal to 200 MHz processor speed, this performance is higher than similar microcontrollers for the same price.

**Figure 5:**
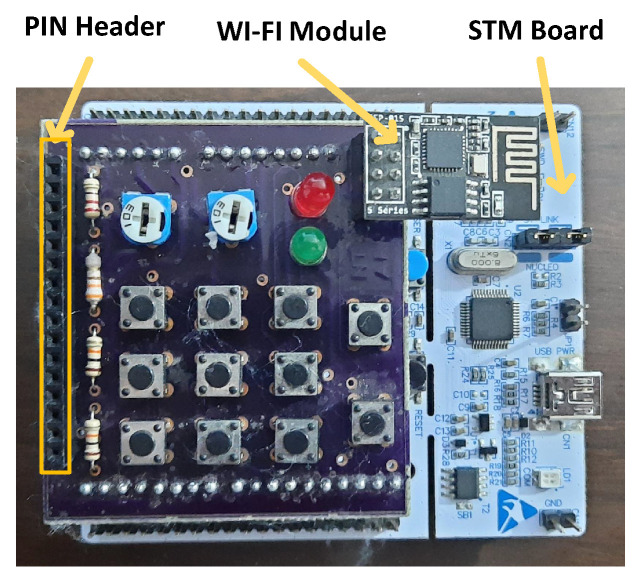
The Proposed CIoT Hardware Unit.

A CIoT hardware platform was implemented as shown in Figure 5 to meet the proposed solution. The platform has three main parts; a WI-FI module for IoT MQTT connectivity, a PIN header for sensors’ interfacing, and a cortex microcontroller for the firmware implementation of the MLP-NN Model and the IoT MQTT client. It is based on the STMicroelectronics (STM) Board. An STM32F401RE microcontroller (MCU) is the main MCU for this board. It has an ARM Cortex-M4 32-bit processor architecture. This processor is a 32-bit processor. It has memories of 512 KBs FLASH and 96 KBs SRAM with a processor speed of up to 84 MHz as a maximum processor speed that is a very low speed with respect to normal PCs or server machines.

The firmware (embedded software) is designed and implemented for the MLP model of the smart device solution. This model is not implemented in case of the cloud-based solution. Steps of the MLP-NN model Implementation are as following;

- Dataset was collected from experts in the automotive field.
- 80% from the data-set was used in TensorFlow training to obtain a specific error and the other 20% for testing the MLP-NN.
- The weights’ matrix *W* was Obtained from the trained MLP-NN TensorFlow model.
- The firmware obtains data from sensors as input neurons. It has the weights’ matrix *W* of the TensorFlow MLP-NN model.
- Algorithm 1 was implemented on the microcontroller in a function called “takeDecision(…)” to obtain the decision.
- The decision is published to the interested entities through the proposed IoT system.

### 4.3. IoT Implementation

The IoT system is implemented for the two solutions, but for the smart device solution, decisions are sent through the IoT system. On the other hand, the sensor data is sent in the cloud-based solution.

After implementing the MLP-NN model on the MCU, then the IoT implementation is required to publish the result from the decision taken as an output from the MLP-NN stage. IoT has different communication protocols, the MQTT protocol is used in this research. It is based on TCP as being connection oriented, which can guarantee a higher level of message delivery.

**Figure 6:**
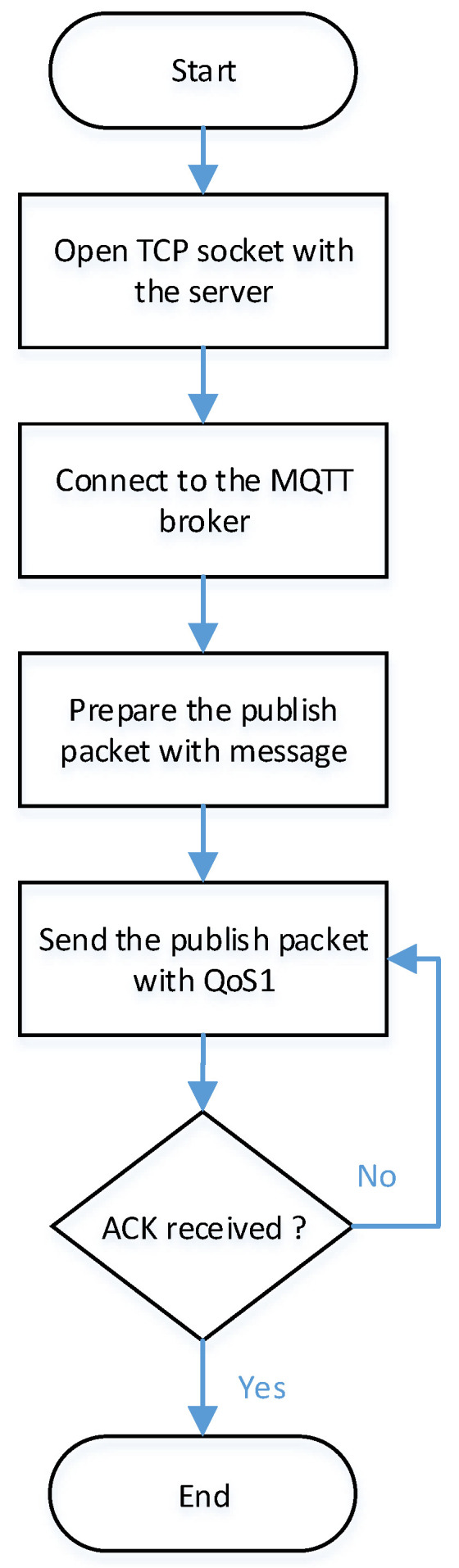
Flow chart of IoT Implementations.

As shown in Figure 6, a TCP connection should be established at first, with an MQTT broker with the server domain name and port. Now, a TCP socket is opened with the server that has the MQTT broker installed. The next step is to send an MQTT connection establishment packet (CONNECT packet). It includes a client identification, and some of connection parameters and flags.

When an MQTT connection is created, the MCU will become an MQTT client. It can publish to any topic on the MQTT and receive any messages through a subscription packet to the MQTT broker. After preparing the message that contains a result of the MLP-NN model, it will be published to a certain topic to the maintenance center. There are different quality of services (QoS) levels; QoS0 means fire and forget, QoS1 denotes at least once of message delivery, and QoS2 guarantees exactly once of message delivery. Generally, Embedded Systems QoS0 and QoS1 are almost used due to lower overhead than the QoS2.

### 4.4. AI and IoT Integration

The AI and IoT are integrated in the smart device solution on the same target hardware. However, the AI is not implemented on the same target device for the cloud based system.

Now, the MLP-NN module, and the IoT MQTT client are implemented on the MCU. In this part, the integration of these parts will be discussed. Due to a successful integration, and due to the hardware limits, a real-time operating system (RTOS) is used. The RTOS is an embedded operation system for multi-tasking.

**Figure 7:**
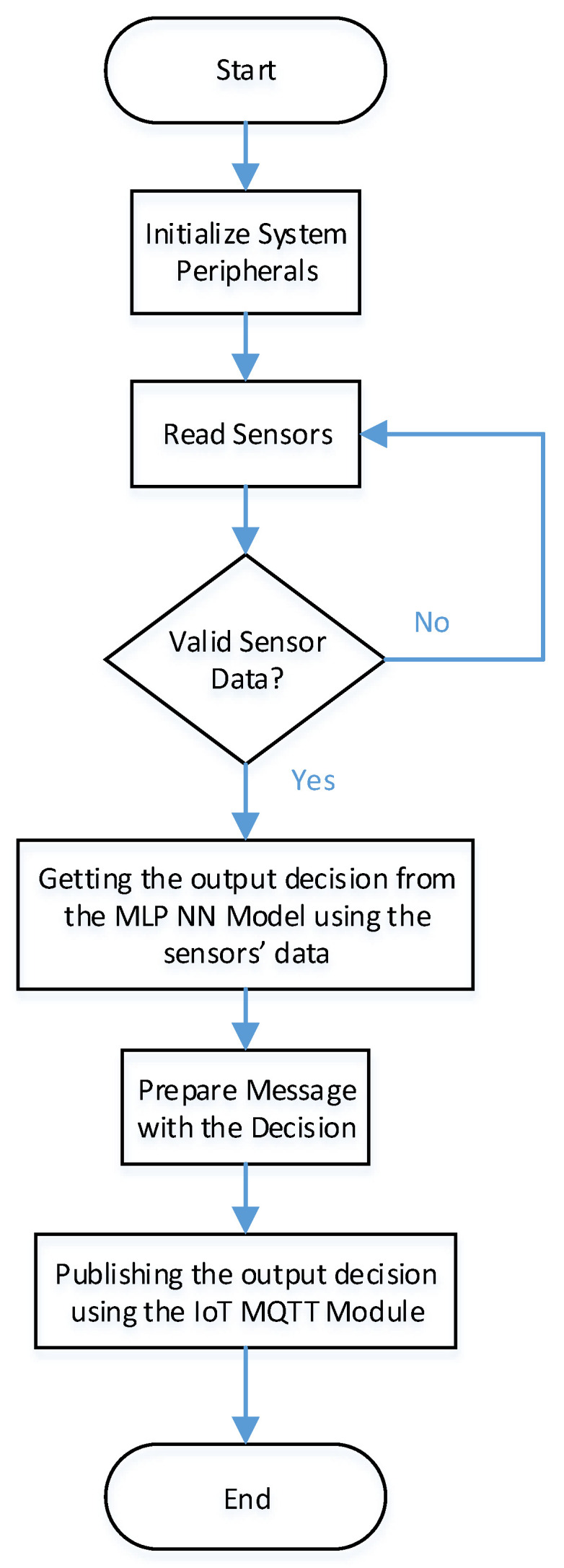
Flow chart of AI-IoT Integration.

As shown in Figure 7, the main firmware will initiate the main MCU peripherals. An RTOS task is created to read the sensors connected to the system. It will validate the sensor readings. It will send the sensor’s valid readings to the MLP RTOS task that is used to process the MLP-NN Model. The MLP task will obtain a decision and prepare a message. It will send the message to the RTOS IoT task to publish the message.

### 4.5. The IoT Communication

There are many IoT communication protocols; the Constrained Access Protocol (CoAP), Message Queuing Telemetry Transport (MQTT), and others. CoAP is working on the UDP transportation protocol unlike the MQTT uses TCP protocol, which is more reliable than the UDP protocol. The proposed device requires sending the diagnostic data to the management center. This data is obtained from the output of the machine learning process. It is transmitted using MQTT. The MQTT protocol is used in embedded systems as it is a lightweight protocol. A real-time operating system (FreeRTOS) is used with the proposed device for multi-tasking purpose. It makes the software more reliable and scalable. An ARM-Cortex M4 processor is commonly used in embedded systems due to its features. It is enhanced to use STMicroelectronics and provides STM32F microcontroller series that are based on ARM-Cortex M4 architecture. It is used in the proposed platform.

An IoT MQTT client requires connecting to the broker by sending a CONNECT packet showing the client identifier. Clients can exchange messages between them. A transmitter client publishes to a specific topic and the receiver node subscribes to the same topic of the publisher node to receive the published messages to this topic. The IoT communication is implemented by developing the MQTT standard protocol with the important packets. A WI-FI hardware module is used for the Internet communication to communicate with the maintenance center publishing a request for maintenance.

The proposed system firmware that is implemented on the MCU reads the current sensor readings and states. It applies these readings as an input to the built MLP-NN system to obtain the result that is one of the model outputs. The firmware decodes the output to a decision to make or an alarm to send to the owner and the maintenance system through the IoT implemented system with the MQTT standard.

## 5. Results

In this section, the training and testing processes were performed completely with the TensorFlow using a dataset that is described in a previous subsection. The single and double hidden layer MLP-NN were implemented to choose the accurate model. Two solutions were implemented in a real network to specify which is better for what. This will be shown in the following subsections.

### 5.1. The Training and Testing of the MLP Neural Network

**Figure 8:**
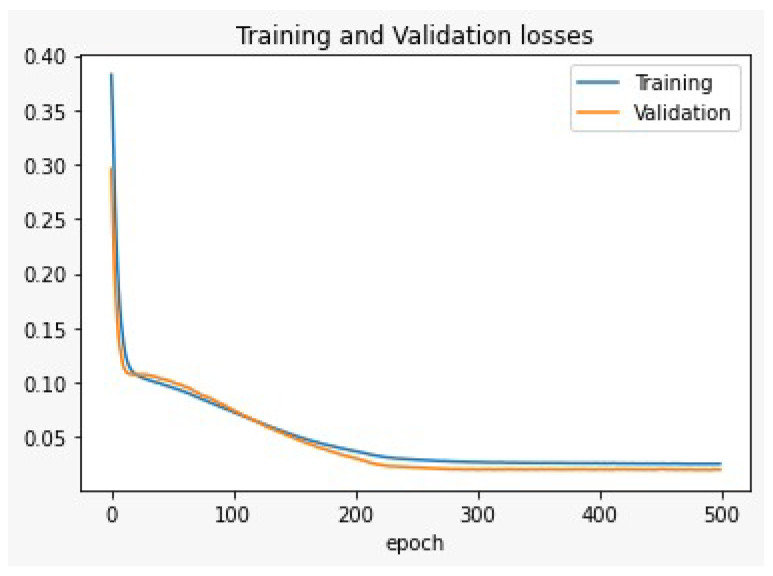
The training and validation losses for the single hidden layer deep learning (MLP).

**Figure 9:**
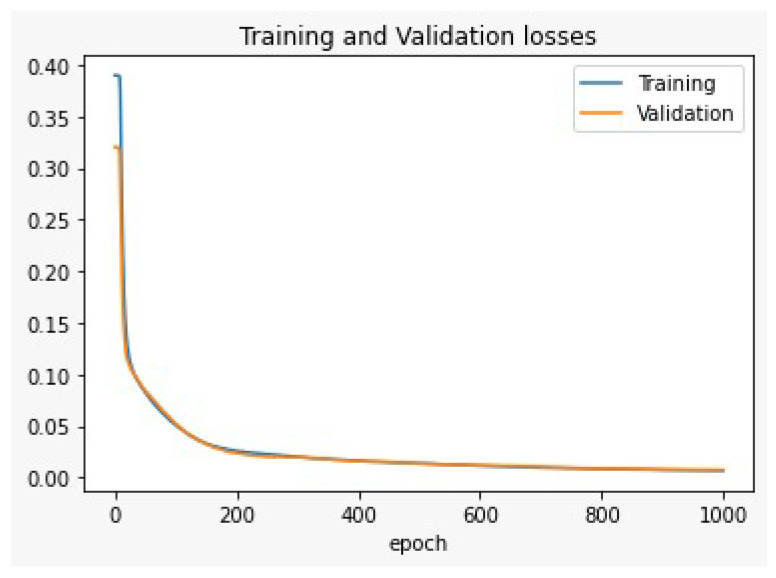
The training and validation losses or the double hidden layers deep learning (MLP).

To select the suitable number of hidden layers in the MLP-NN structure in our model, we divided the main dataset into training and testing datasets. Then, the training dataset (80% of the main dataset) was used to train the model, and the testing dataset (20% of the main dataset) was used to test the model using Keras/Tensorflow deep learning platform. The metric that was used to validate the results was Mean Square Error (MSE), which called training and validation losses shown in Figure 8 and Figure 9. As shown in Figure 8, the training and validation results are not very promising for the MLP-NN single hidden layer structure. That model needs a lot of iterations (epochs) to decrease the losses during training and testing phases. So, we increased the hidden layers to be double hidden layers MLP-NN. Then, by using the same training and testing datasets, the training and validation losses enhanced as shown in Figure 9. From these results, the double hidden MLP-NN is suggested for the proposed CIoT system since MSE error of the double hidden layer is better than the single hidden layer MLP-NN.

### 5.2. Proposed Solutions Comparative Study

In this section, a study and a discussion are carried out with results for the two proposed solutions; the smart device solution that has the implementation of the MLP-NN model, and the cloud-based MLP-NN model. To study the proposed solutions’ performance, two performance metrics were chosen in the experiments: the delay (i.e., latency), and the total transmitted bytes. The delay is defined as the interval time between gathering the sensors’ data and receiving the taken decision by the maintenance center. While total transmitted bytes are the total number of bytes that are transmitted per a successfully sent decision message.

**Figure 10:**
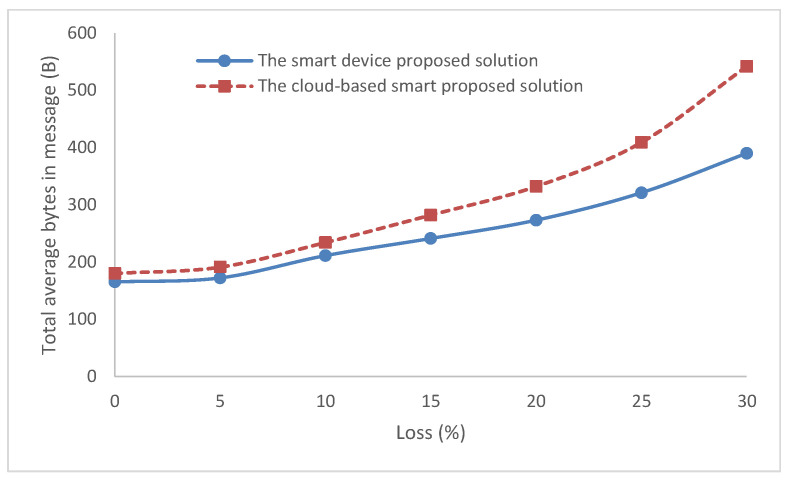
The total average bytes in message.

The smart device takes the sensors’ data and applies it to the implemented MLP model to obtain the output decision. Conversely, if the system sends this data to the server to have the decision back, this will require more traffic. Therefore, the proposed smart device has lower traffic than the cloud-based architecture as shown in Figure 10 and Table 2. The traffic difference in bytes increases with more network loss. It is 15 bytes with no losses and increases with more loss in the network until it reaches 152 bytes at 30% network loss.

**Figure 11:**
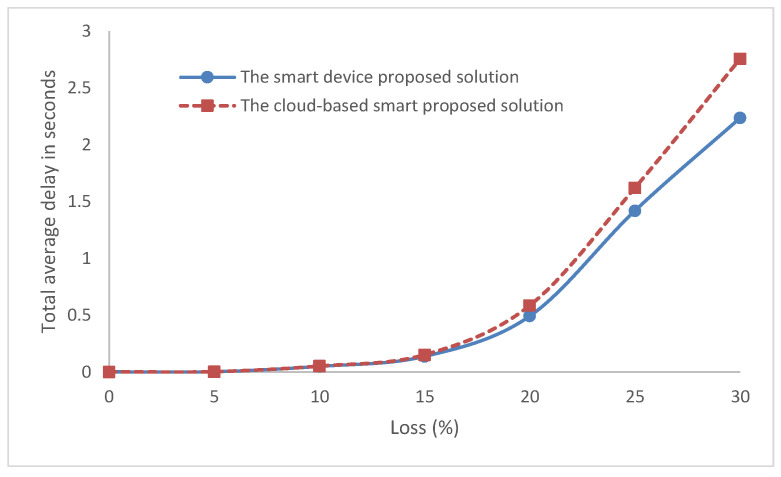
The total average delay for message.

Moreover, as shown in Figure 11 and Table 3, when loss increases in networks, the proposed smart device has lower delays than the proposed solution of the cloud-based system. The delay difference was significant from 15% and above in the lossy network.

However, the server-based system is simple to re-train, as the smart devices require training on another entity and then re-updating the MLP model. In addition, updates are easier to update on one device in the case of the cloud model rather than the smart device structure.

We can summarize the pros and cons of the On-Cloud training process and the On-Device training process as shown in Table 4.

In Table 5, a summary is introduced to the pros and cons for the On-Cloud operation process and decision maker, and the On-Device operation process and decision maker.

## 6. Conclusions

In this paper, a smart cognitive IoT devices solution that integrates the AI and IoT system was introduced in the industry automotive field. It has an AI implementation of the MLP-NN with the double hidden layer and the single hidden layer. As a result, the double hidden layer was better in terms of testing and validation accuracy. After that, two solutions were proposed: the smart device solution, which uses the implementation of the MLP-NN model on ARM-Cortex as a limited microcontroller; and the other solution is the cloud-based MLP-NN model, which is implemented on the cloud AI server.

For the first proposed solution, the hardware implementation of the proposed MLP-NN is successfully integrated with the implementation of IoT MQTT and real sensor interfaces on an ARM-Cortex as an example of a limited microcontroller.

A comparison of the two solutions with parameters such as the delay and throughput were performed. The cloud-based solution required more traffic and delay than the smart device solution. The delay difference was bigger from 15% and above in the lossy network. The difference in traffic bytes was 15 bytes at first with no losses and increased with the increasing of the network loss until it reached 152 bytes at 30% network loss. However, the cloud-based system was simple to re-train, as the smart devices required training on another entity and then the re-update was carried out for the MLP model. In addition, updates were easier to be performed on one device in the case of the cloud model rather than the smart device solution.

## Figures and Tables

**Table 1:** The relevant work comparison.

Related Work	Training Process	Operation Process	Decision Maker
[8,9,21,24,25,26]	on-cloud	on-cloud	on-cloud
[10,11,16,18,19,20,23]	on-device	on-device	on-device
The proposed solution	on-cloud	on-device	on-device

**Table 2:** The total average bytes in message for the two solutions with network loss.

Loss	The Smart Device Proposed Solution	The Cloud-Based Smart Proposed Solution
0	165	180
5	172	191
10	211	234
15	241	282
20	273	332
25	321	409
30	390	542

**Table 3:** The total average delay (seconds) for message for the two solutions with network loss.

Loss	The Smart Device Proposed Solution	The Cloud-Based Smart Proposed Solution
0	0.001012	0.001035
5	0.002031	0.002291
10	0.049147	0.051023
15	0.137203	0.151072
20	0.492318	0.585108
25	1.420015	1.621284
30	2.237014	2.758305

**Table 4:** The pros and cons of the On-Cloud training process and the On-Device training process.

	On-Cloud Training Process	On-Device Training Process
Pros	- Cloud powerful training (No limited learning)	- Secure (local processing)
	- Easy to do	- Internet connectivity is not required
	- Hardware Independent	
	- Low-cost training	
	- No embedded expensive hardware is required	
Cons	- Security and privacy issues	- Limited learning compared to the Cloud learning
	- Internet connectivity is required	- Requires high-capable devices (memory and speed)
		- Hardware dependent
		- Expensive due to hardware cost

**Table 5:** The pros and cons of the On-Cloud operation process and decision maker, and the On-Device operation process and decision maker.

	On-Cloud Operation Process and Decision Maker	On-Device Operation Process and Decision Maker
Pros	- Training and operation process on the same machine	- No further delay is required for decision-making
	- Any update on MLP-NN structure is easier to rebuild	- Secure enough due to local actions
	- Faster powerful computing	- Internet connectivity is not required
	- Model updates on cloud only	- Works on limited-resources microcontrollers
Cons	- Higher delay to get the decision back from the Cloud	- Requires updating for any MLP-NN structure
	- Internet connectivity is required to take the decision	- Model updates should be applied to all devices
	- Security and privacy issues	

## Data Availability

Not applicable.
